# Maternal and fetal recovery after severe respiratory failure due to influenza: a case report

**DOI:** 10.1186/1756-0500-6-62

**Published:** 2013-02-15

**Authors:** Kristine Madsen, Ditte Gry Strange, Morten Hedegaard, Elisabeth R Mathiesen, Peter Damm

**Affiliations:** 1 Department of Obstetrics, Center for Pregnant Women with Diabetes, The Juliane Marie Centre, Rigshospitalet, Faculty of Health Sciences, University of Copenhagen, Copenhagen, Denmark; 2 Department of Anesthesiology, Center for Pregnant Women with Diabetes, The Juliane Marie Centre, Rigshospitalet, Faculty of Health Sciences, University of Copenhagen, Copenhagen, Denmark; 3 Department of Endocrinology, Center for Pregnant Women with Diabetes, The Juliane Marie Centre, Rigshospitalet, Faculty of Health Sciences, University of Copenhagen, Copenhagen, Denmark

**Keywords:** Pregnancy, Diabetes, Influenza, Pneumonia, Mechanical ventilation, Pregnancy outcome

## Abstract

**Background:**

During pregnancy women are at increased risk of severe complications to influenza infection, including death of mother or fetus, especially if chronic comorbid medical conditions such as diabetes mellitus are present.

**Case presentation:**

A 36 years old Caucasian pregnant woman with type 1 diabetes underwent mechanical ventilation in gestation week 27 for severe respiratory failure due to influenza and pneumonia. For three weeks during and following her most severe illness, fetal growth could not be detected and the umbilical flows and amniotic fluid volumes were affected too. The possibility of preterm delivery and extracorporeal membrane oxygenation (ECMO) treatment were considered, however the patient and her fetus recovered gradually on conservative treatment. Under close surveillance the pregnancy continued until term, with delivery of an infant with appropriate weight for gestational age.

**Conclusion:**

Preterm delivery and decreased birth weight were reported for women with antepartum pneumonia. Mechanical ventilation and ECMO treatment for severe respiratory failure in pregnancy are life threatening conditions and have been associated with preterm delivery. It remains uncertain if delivery improves the respiratory status of a critically ill woman, and the fetal condition is likely to improve, if the maternal condition is stabilized.

Severe respiratory insufficiency requiring mechanical ventilation in a diabetic pregnant woman with influenza was successfully treated conservatively. Despite clear signs of impaired fetal condition in the acute phase, watchful waiting resulted in delivery of a normal weight infant at term.

## Background

Viral influenza is caused by either Influenza A or B, and both types may cause significant morbidity and mortality. Pregnant women in advanced stages of pregnancy are at increased risk of serious infection, hospitalization and death. Smoking and preexisting chronic medical conditions, including diabetes, predispose to serious illness [1-7].

Pulmonary complications are the most frequent and include secondary bacterial pneumonia. The complicating pathogens are often *Streptococcus pneumonia*, *Staphylococcus aureus* and *Haemophilus influenza*[2,8]. Influenza and pneumonia may be complicated by severe respiratory failure necessitating mechanical ventilation and extra corporeal membrane oxygenation (ECMO) treatment [7,9,10]. Antiviral therapy (e.g. oseltamivir) should be started within 48 hours of symptom onset for best effect. Delayed administration (after 48 hours) is associated with increased intensive care unit (ICU) admission and higher maternal mortality [11-14]. Secondary bacterial pneumonia should be treated promptly with antibiotics [2].

We report a unique case where a pregnant woman with diabetes underwent mechanical ventilation for severe respiratory failure due to influenza and pneumonia. During and following her most critical and severe illness, her fetus did not grow for several weeks. Both the woman and her fetus recovered, and the pregnancy was continued until term with a good outcome for both mother and child.

## Case presentation

### Maternal condition

Our patient was a 36 years old Caucasian pregnant woman with type 1 diabetes mellitus for 22 years complicated with proliferative retinopathy, but no hypertension or microalbuminuria. HbA1c was 6.6% at the first pregnancy visit (upper normal range in pregnancy is 5.6% [15]). She smoked 20 cigarettes daily, but had no known pulmonary disease. The patient had been pregnant once previously, where she delivered a healthy girl at term by elective cesarean section.

The current pregnancy was uneventful until she presented at gestational age (GA) 26 weeks and 1 day (26+1) at the Department of Obstetrics. She had been ill for three days, and demonstrated a productive cough, high fever, dyspnea and increased respiratory frequency. Capillary oxygen saturation was 83%, and bilateral lung infiltrations were seen on chest x-ray. Influenza A (H1N1) and pneumonia were suspected, and antiviral therapy (oseltamivir) and broad-spectrum intravenous antibiotics (cefuroxim and clarithromycin) were started. Two doses of betamethasone were administered for fetal lung maturation.

During the next day respiratory function deteriorated progressively, and the patient was transferred to the ICU, where she was intubated and mechanical ventilation initiated. Her condition was considered life threatening with severe acute respiratory distress syndrome and progressive hypoxemia despite ventilator treatment. Termination of pregnancy was considered to optimize ventilation; however maternal ECMO therapy was also deliberated and due to possible risk of maternal bleeding, continuation of pregnancy was decided.

The following days the patients’ condition stabilized. She was extubated after 7 days and discharged from the ICU at GA 27+6. She continuously had a severe productive cough and needed nasal oxygen supplementation. A thoracic CT demonstrated bilateral multiple pulmonary abscess formations. Broad-spectrum intravenous antibiotics were administered for another four weeks, during which the patients’ clinical condition improved gradually. Microbiologic examinations showed positive Influenza B polymerase chain reaction (PCR) in tracheal secretions and *Staphylococcus aureus* in expectorate and larynx secretion samples.

**Figure 1:**
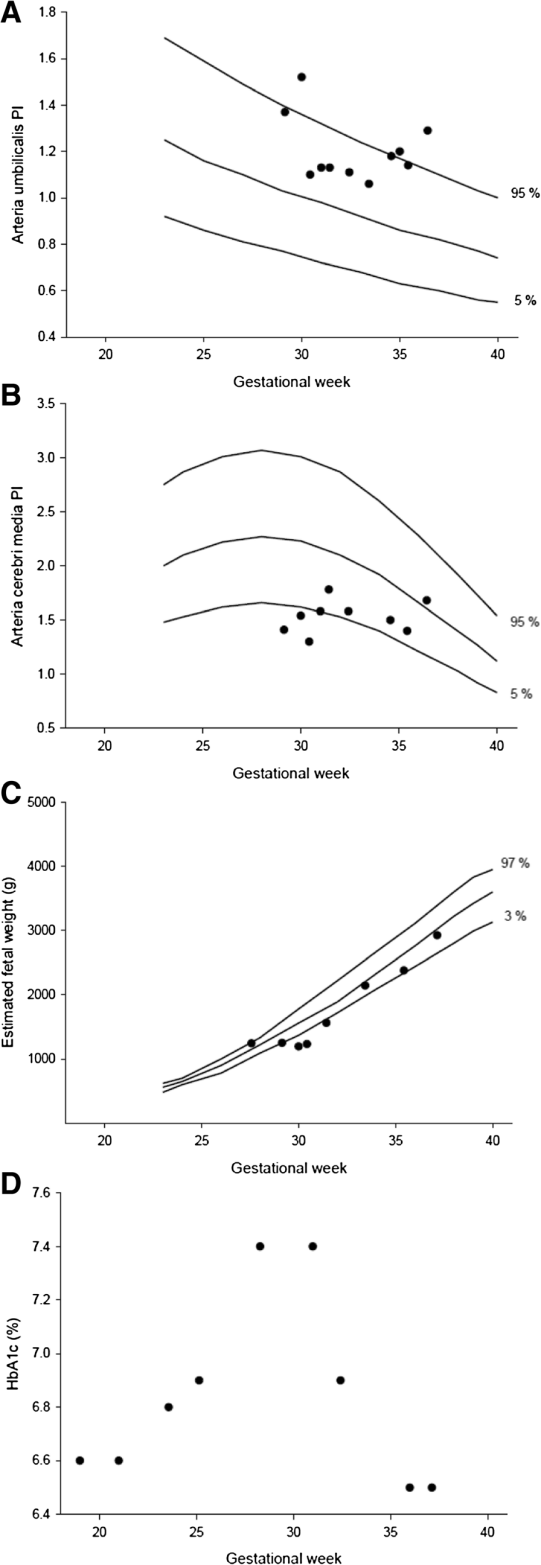
**Graphical illustration of the association between estimated fetal weight, HbA1c and flows in a. umbilicalis and a. cerebri media measured during our patients’ pregnancy.** Reference values from Astraia Obstetric and Gynecological database are included [16]. Upper normal range for HbA1c in pregnancy is 5.6% [15].

The patients’ blood glucose levels were difficult to control during the most critical weeks and included both hypoglycemic episodes and one hyperglycemic period with mild ketoacidosis. Hb1Ac values are shown in Figure 1.

### Fetal condition

It was decided to prioritize the mother, and initially the fetal condition was only monitored by checking the heart rate daily with ultrasound. A fetal ultrasound scan was performed at GA 27+ 4, with a normal estimated fetal weight of 1242 g (+ 4.5% above the mean) and normal amniotic fluid suggesting an unaffected fetus. However 3 weeks later at GA 30+3 fetal weight was estimated to 1231 g (− 29.9% below the mean), indicating impaired fetal growth for the prior three weeks. This was further supported by increased pulsatility index (PI) in a. umbilicalis, reduced amniotic fluid volume and abnormal flow in a. cerebri media (brain sparring), see Figure 1.

The patient and her husband were informed that preterm delivery could be necessary if fetal conditions deteriorated further. Fortunately at GA 31+3 the fetus had grown, with an estimated fetal weight of 1561g (− 16.6% below the mean). The amniotic fluid volume and the flows in a. umbilicalis and a. cerebri media had normalized. The fetus continued to grow appropriately, and was born by elective cesarean section at GA 37+1 weighing 2924 g (− 3.5% below the mean). The newborn needed neonatal assistance for a few days due to intermittent apnea, low blood glucose and jaundice. Both mother and daughter were discharged from the hospital eight days post partum.

## Discussion

One in 1000 healthy pregnant women have been estimated hospitalized yearly because of influenza in Canada [17]. During the 2009 H1N1 influenza pandemic in the U.S.A. the hospitalization rate among pregnant women was approximately four times the rate in the general population [12,13].

The U.S.A. Centers for Disease Control and Prevention reported a 20% mortality rate for H1N1 infected pregnant women admitted to the ICU during the 2009 H1N1 pandemic. Overall 3.8% of the reported infected pregnant women died [14]. A mortality rate of 8% was reported among infected pregnant and postpartum women in California during the 2009 H1N1 pandemic [13]. The mortality for ICU-admitted pregnant women presenting with primary viral pneumonia was 14% during the 2009 H1N1 pandemic in Spain [18].

Decreased birth weight and preterm delivery of infants born to women with antepartum pneumonia were described in several studies [19-21], but not all [6]. The risk of preterm birth was furthermore increased when women with pneumonia had another underlying comorbid condition [7]. Fetal outcome of pregnant women admitted to the ICU for non-obstetric causes, included 34% fetal losses and 11% of the infants needed neonatal intensive care [22].

The maternal mortality rate in obstetric patients undergoing mechanical ventilation was 14% [23]. Successful maternal outcome after ECMO treatment for severe respiratory failure/acute respiratory distress in pregnancy was reported in small series [8-10,24-26]. It has been necessary to administer systemic anticoagulation to enable ECMO treatment; hence hemorrhagic complications have been common with reported rates of 54% among pregnant and non-pregnant patients [8]. In most prior case reports preterm delivery was reported for women undergoing mechanical ventilation or ECMO treatment due to severe respiratory failure [3,9,10,24,25,27]. A concerning fetal condition is likely to improve, if the maternal condition is stabilized [28].

Limited publications have reported that delivery could improve the respiratory status of a critically ill pregnant patient. Reduced oxygen requirement was demonstrated after delivery in respiratory compromised patients, but still no clear benefit of delivery was established when attempting to improve maternal oxygenation [29].

The Royal College of Obstetricians and Gynaecologists have not recommended termination of pregnancy due to the need for ECMO treatment in pregnancy. Delivery should be to facilitate mechanical ventilation, allow for further intervention or when fetal compromise was evident. Before 30–32 weeks, the size of the uterus should not affect mechanical ventilation significantly [30].

## Conclusion

The woman in our case had diabetes and was a heavy smoker. She developed influenza complicated by secondary bacterial pulmonary infection requiring mechanical ventilation in late pregnancy. The fetus demonstrated impaired growth and abnormal flows in a. umbilicalis and a. cerebri media during the critical period of the disease. This case was significant as the patient continued her pregnancy under close surveillance despite these high risk conditions. Both the mother and fetus survived with an infant delivered at term and appropriate in size.

## Consent

Written informed consent was obtained from the patient for publication of this Case report and any accompanying images. A copy of the written consent is available for review by the Series Editor of this journal.

## Abbreviations

ECMO: Extra corporeal membrane oxygenation;ICU: Intensive care unit;HbA1c: Hemoglobin A 1c;GA: Gestational age;PCR: Polymerase chain reaction;PI: Pulsatility index

## Competing interests

The authors declare that they have no competing interests.

## Authors’ contributions

DGS, PD and KM contributed to the case report design, and collected and critically reviewed the relevant literature. DGS, ERM, MH, PD and KM analyzed and discussed the patient’s case critically using their special clinical and scientific skills from the fields of obstetrics, diabetology and anesthesiology. KM wrote the first draft of the case report. Hereafter all authors (DGS, ERM, MH, PD and KM) participated in the review process with critical revision, discussion and optimization of the manuscript. DGS, ERM, MH, PD and KM all approved the final version of the manuscript.
